# Drastic Microbial Count Reduction in Soy Milk Using Continuous Short-Wave Ultraviolet Treatments in a Tubular Annular Thin Film UV-C Reactor

**DOI:** 10.3390/foods12203813

**Published:** 2023-10-17

**Authors:** María Martínez-García, Jezer N. Sauceda-Gálvez, Idoia Codina-Torrella, María Manuela Hernández-Herrero, Ramón Gervilla, Artur X. Roig-Sagués

**Affiliations:** 1 Centre d’Innovació, Recerca i Transferència en Tecnologia dels Aliments (CIRTTA), TECNIO CERTA-UAB, Departament de Ciència Animal i dels Aliments, Universitat Autònoma de Barcelona, Travessera dels Turons S/N, 08193 Barcelona, Spain; martinez_maria91@hotmail.com (M.M.-G.); jezer_noe@hotmail.com (J.N.S.-G.); manuela.hernandez@uab.cat (M.M.H.-H.); 2 Departament d’Enginyeria Agroalimentària i Biotecnologia, Edifici D4C, Esteve Terradas, 8, 08860 Castelldefels, Spain; idoia.codina@upc.edu; 3 SPTA-Servei Planta Tecnologia Aliments, Universitat Autònoma de Barcelona, c/de l’Hospital S/N, 08193 Barcelona, Spain; ramon.gervilla@uab.cat

**Keywords:** *Listeria monocytogenes*, *Escherichia coli*, *Bacillus subtilis*, *Aspergillus niger*, soy milk, short-wave ultraviolet (UV-C)

## Abstract

Vegetative cells of *Listeria monocytogenes* and *Escherichia coli* and spores of *Bacillus subtilis* and *Aspergillus niger* were inoculated in soy milk at an initial concentration of ≈5 log CFU/mL. Inoculated and control (non-inoculated) soy milk samples were submitted to three types of treatments using a tubular annular thin film short-wave ultraviolet (UV-C) reactor with 1 mm of layer thickness. Treatments applied depended on the flow rate and the number of entries to the reactor, with UV-C doses ranging from 20 to 160 J/mL. The number of entries into the reactor tube (NET) was established as the most determining parameter for the efficiency of the UV-C treatments. Conidiospores of *A. niger* were reported as the most resistant, followed by *B. subtilis* spores, while vegetative cells were the most sensible to UV-C, with *Listeria monocytogenes* being more sensible than *Escherichia coli.* Treatments of just 80 J/mL were needed to achieve a 5 log CFU/mL reduction of *L. monocytogenes* while 160 J/mL was necessary to achieve a similar reduction for *A. niger* spores.

## 1. Introduction

The consumption of soy products has increased in the last years due to the health benefits of soybean compounds, such as reduced LDL cholesterol and protection against osteoporosis [1]. Soy milk is the liquid extract obtained by mixing and grounding soybeans with water, resulting in a product with a similar appearance to cow’s milk, although with a different composition [2]. Commercial soy milk is usually stabilized using thermal methods. These processes may cause undesirable changes in nutritional value, such as the destruction of essential amino acids and vitamins, as well as modification of sensorial properties [3,4].

Nonthermal technologies, such as short-wave ultraviolet (UV-C) radiation, are currently being investigated as an alternative to heat pasteurization processes. UV-C showed good capability to reduce or eliminate spoilage and pathogenic microorganisms in foods [5]. The application using reactors that allows for processing fluid foods in a continuous process increased interest in this technology as an alternative to thermal treatments, as it is easy to use, does not generate chemical residues, and can be effective at a lower cost [6,7]. Moreover, it preserves the nutritional and quality properties of foods better [8]. UV-C radiation forms dimers of pyrimidine in the exposed microorganisms, blocking DNA duplication what kills microbial cells [9,10], including bacterial spores that cannot be eliminated using other nonthermal technologies [11,12].

The microbiocidal effect of UV-C has been evaluated in different types of food, such as fruit juices, vegetable smoothies, liquid egg, and milk, demonstrating effectiveness similar to thermal pasteurization [13,14,15,16]. Few studies have been conducted on soy milk, although the potential of this technology to eliminate both vegetative and sporulating bacteria has been demonstrated [17,18]. However, soy milk presents a high UV-C absorption coefficient, which is a limiting factor that reduces the possibility that UV-C radiation reaches all the microorganisms present in the foodstuff and, consequently, treatment effectiveness [19,20]. To increase the efficiency of UV-C treatments using reactors, both the UV-C radiation dose and the flow pattern (laminar flow or turbulent flow) must be correctly adjusted. The absorbed UV-C radiation dose is influenced by both the distance from the source of the UV-C radiation during exposure and the presence of food particles [8], but the presence of food particles may also interfere. Different designs of continuous flow UV-C reactors have been described in the literature. Recently, Hirt et al. [21] compared the overall efficacy of inactivation in *Saccharomyces cerevisiae* between different designs of UV-C reactors (annular thin film, serpentine, and coiled tube reactors), concluding that the inactivation was less efficient when a laminar flow was generated in the reactor since vortices are needed to interchange the portion of the volume directly facing the UV-C source. Another proposed strategy is to reduce the space between the UV-C source (lamp) and the farthest particle of the matrix, reducing the path length by creating a thin film around the UV-C source. However, this system makes liquid flow under laminar flow conditions, which is an inconvenience when treating high absorbance coefficient matrices.

Reverter-Carrión et al. [22] reported that in annular thin film UV-C reactors, efficacy against spores of *Bacillus subtilis*, *Geobacillus stearothermophilus*, *Alicyclobacillus acidoterrestris*, and *Aspergillus niger* increased when UV-C treatments were applied through several cycles. This strategy allowed for overcoming the inconvenience of the laminar flow generated in this type of UV-C reactor. More recently, Martinez et al. [13] introduced the concept of the number of entries in the reactor tube (NET), consisting of applying several cycles, as a parameter to establish the efficiency of the UV-C treatments in reactors with laminar flow, reporting that increasing NET improves the efficacy of treatment.

This study aimed to evaluate the effectiveness of UV-C treatments consisting of applying a different number of passes (cycles) to different microorganisms (spores of *Bacillus subtilis* and *Aspergillus niger*, and vegetative cells of *Escherichia coli* and *Listeria monocytogenes*) inoculated in soy milk. To determine the importance of the variables on the efficacy of the treatments, some mathematical models were proposed.

## 2. Materials and Methods

### 2.1. Soy Milk Physicochemical Characteristics

An organic commercial UHT-treated soy milk containing 14% soybean was used to evaluate the lethal effect of the treatments tested. The coefficient of absorbance at 254 nm (AC_254_) was measured with a Nanophotometer Pearl spectrophotometer (IMPLEN, München, Germany), and the result was 500 ± 0.1 cm^−1^. pH was measured directly using a pH meter (pH basic 20, Crison, Barcelona, Spain), giving a mean value of 6.34 ± 0.01; turbidity was measured with a portable EPA 2100Q turbidimeter (HACH, Hospitalet de Llobregat, Spain), and the value obtained was 35,983 ± 0.041 NTU (nephelometric turbidity units). The relative viscosity was obtained using an Ostwald 1293 model viscometer (CIVEQ, Mexico City, Mexico) 1.047 ± 0.003 mPa·s. Density was determined with a hydrometer–aerometer–densitometer HYDR-100-001 (Labbox, Vilassar de Dalt, Spain), and the value obtained was 1.014 ± 0.000 g/mL.

### 2.2. UV-C Equipment

**Figure 1:**
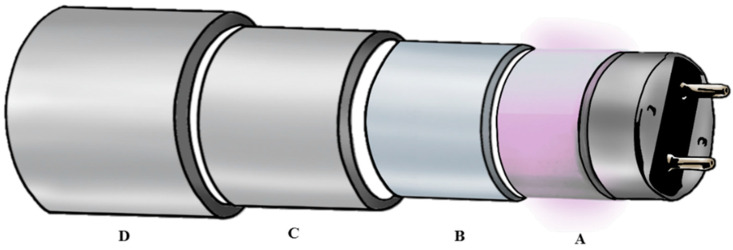
UV-C reactor structure. A: UV-C lamp; B: quartz protection glass; C and D: stainless steel tubes. Section A–B: air circulation; section B–C: food matrix circulation area; section C–D: water cooling system [13].

In this survey, a UV-C reactor was used (European Patent EP 2965766-A1, UV-Therm, Ypsicon S.L., Barcelona, Spain). The reactor consists of two low-pressure mercury UV-C lamps (UV-Consulting Peschl Spain, Castellón, Spain), with 55 W of total electrical power and 41 mW/cm^2^ of irradiance, that are protected by a 2 mm thick quartz tube (UV-Consulting Peschl, Geldo, Spain). The reactor is fed using a peristaltic pump (Flowmaster FMT300, ISMATEC Lab. GmbH, Wertheim-Mondfeld, Germany) connected through a pipe of silicone, having a total volume capacity of 673 mL. Figure 1 shows the different concentric cylinders that forms the UV-C reactor: air circulates between sections A and B to cool the UV-C lamp; food matrix circulates between sections B and C in a layer of 1 mm; water flows between sections C and D to control the temperature of the treatments. In this survey, the temperature was adjusted to 20 °C.

### 2.3. UV-C Treatments

Table 1 shows the features of the treatments applied (UV-C dose, flow rate, retention (exposure) time, Reynolds number, and number of entries in the reactor tube (NET)). All treatments were applied at 20 °C. NET was calculated by the following formula:

(1) $$NET=\frac{{t\cdot L\cdot C}_{A}}{V_{C}}.$$

where *t* corresponds to the treatment time, expressed in seconds; *L* to the number of lamps in the reactor (2); *C_A_* the flow rate at which the matrix circulates, expressed as mL/s; and *V_C_* to the total volume of food circulating through the circuit, expressed as mL (70 mL).

Reynolds number (Re) was determined according to the formula described by Müller et al. [23]:

(2) $$Re=\frac{d\cdot v\cdot \rho }{\eta }$$

where *d* is the diameter of the tube (m), *v* is the velocity (m/s), *ρ* is the fluid density (kg/m^3^), and *η* is the dynamic viscosity of the fluid (Pa·s). Flow is considered laminar when the value of Re is 2100 or lower.

### 2.4. Dosimetry

The UV-C energy emitted by the lamp per volume unit at 254 nm was measured using the chemical iodide/iodate actinometer developed and described by Rahn [24] using a potassium iodide solution passed through the UV-C reactor. Its absorbance was then measured at 352 nm using a Nanophotometer Pearl spectrophotometer (IMPLEN) after diluting the sample with distilled water ten times. The formula proposed by Linden and Mofidi [25] was used to calculate the power of the UV-C reactor lamps:

(3) $$H_{MP}=\frac{\left({\Delta OD}_{352}\right)\left(V\right)\left(23,786.4\right)}{\left(I\right)\left(1+0.02\left(T-20.7\right)\right)A}$$

where *H_MP_* is the applied UV-C dose of the UV-C lamp in mJ/cm^2^; Δ*OD*_352_ is the difference between the absorbance of the irradiated sample and absorbance of the untreated (white) sample measured at 352 nm; *V* is the volume of the irradiated sample in L; *I* is the path length of light passing through the solution in cm (in the reactor used, it was 0.1 cm); *T* is the temperature of the treatment, expressed in °C; *A* is the area of sample facing the light source (700 cm^2^); and 23,786.4 is the constant (mJ/cm^2^) specific for LMP-UV-C lamps.

### 2.5. Effective Depth of UV-C

To determine the depth at which the action of the UV-C_254 nm_ radiation was most effective (lethal zone), the Lambert–Beer equation was applied:

(4) $$I=I_{0}\cdot e^{-k\cdot c\cdot d}$$

where *I* and *I*_0_ correspond to the intensity of the UV-C radiation, expressed in mW/cm^2^; *I* corresponds to the intensity that the matrix receives at a given point and I_0_ corresponds to the intensity emitted by the reactor. The constant *k* corresponds to the AC_254_ of the matrix, expressed in cm^−1^; *c* is the concentration of solutes capable of absorbing UV-C of the sample, expressed in mol/L; finally, *d* is the depth or distance at the must UV-C light passes through expressed in cm. According to Koutchma [19], to inactivate microorganisms, they must be exposed to a dose of at least 1 mJ/cm^2^ to cause irreversible genetic damage, and consequently, the limit of the effective distance is where the dose achieved is 1 mJ/cm^2^. The width of the lethal zone varies depending on the matrix and its absorbance. For determining the exposed dose, a particle size of 1 μm and a matrix pass of 1000 μm were considered to determine this zone. Moreover, the number of times that a particle passes through the lethal zone (NEL value) was calculated from the probability that any particle of the matrix passes through the lethal zone using the following formula:

(5) $$NEL=\;\frac{d}{AT}\cdot NET$$

where *NET* is the number of entries in the reactor tube, *d* is the width of the lethal zone, expressed in µm, and *AT* is the width of the space through which the matrix passes, expressed in µm (in that case, 1000 μm). The ratio *d*/*AT* is the probability that a particle can enter the lethal zone.

### 2.6. Preparation of Microbial Strains and Inoculation in Soy Milk Samples

Strains of *Escherichia coli* (CECT423), *Listeria monocytogenes* (CECT4031), *Bacillus subtilis* (CECT 4002), and *Aspergillus niger* (CECT 2574) were obtained from the Spanish Type Culture Collection (CECT, Universitat de València, Valencia, Spain) as a freeze-dried culture. *E. coli* and *L. monocytogenes* were first recovered in tryptone soy broth (Oxoid, Basingstoke, UK) incubated overnight (18–20 h) at 37 °C and later streaked on a Petri plate with tryptone soy agar (Oxoid, Basingstoke, UK) and incubated overnight (18–20 h) at 37 °C. After testing the purity of cultures, some colonies were transferred to 0.9% (*w*/*v*) saline solution, and density was adjusted to a number 1.0 of McFarland scale for *L. monocytogenes,* and 1.5 for *E. coli* (around 10^8^ CFU/mL) with a Densimat (Biomérieux, Crappone, France).

A modification of the UNE EN 13704:2019 procedure [26] was used to obtain the *B. subtilis* spores suspension. Briefly, the lyophilized culture was rehydrated in 10 mL of glucose and tryptone broth (TGB: 2.5 g of yeast extract (Oxoid), 5 g of tryptone (Oxoid), 1 g of glucose (Sigma-Aldrich, St. Louis, MI, USA), in 1 L of distilled water, pH adjusted to 7.2), and incubated for 24 h at 30 °C. Two milliliters of this culture was transferred to Roux bottles containing yeast extract agar (MYA: 10 g of meat extract (Oxoid), 2 g of yeast extract (Oxoid), 15 g of agar (Oxoid), and 0.04 g of MnSO_4_·H_2_O (Merck, Darmstadt, Germany) in 1 L of distilled water), which were incubated at 30 °C for up to 30 days. The formed spores were collected by adding 20 mL of sterile distilled water to the Roux bottles and scraping the surface with a Digralsky stick. Spore suspensions were pooled and washed four times in 15 mL cold sterile water by centrifugation at 10,000× *g* for 20 min at 4 °C using a Sigma 4K15 centrifuge (Sigma Laborzentrifugen GmbH, Osterode am Harz, Germany). The resulting sediment was then suspended in 30 mL of sterile distilled water and subjected to heat treatment at 75 °C for 10 min to ensure the inactivation of vegetative cells. The resulting spore suspension was stored at 4 °C until use. 

A modification of the UNE-EN 1650:2020 standard procedure [27] was used to prepare the *A. niger* spore suspensions. The freeze-dried culture was rehydrated in 9 mL of tryptic soy broth (TSB) (Oxoid) and incubated at 30 °C for 24 h. This cultured broth was used to inoculate Petri dishes with potato dextrose agar (PDA) (Oxoid) and was incubated at 30 °C for 7 days. After that, 3 mL of nutrient broth (NB) (Oxoid) were added to the Petri dishes, and the surface was scraped out with a Digralsky stick. One milliliter of the resulting suspension was transferred into 9 mL of NB and incubated at 30 °C for 24 h. Subsequently, 2 mL of this culture was inoculated into Roux flasks containing PDA and incubated at 30 °C until the culture color changed to black (maximum sporulation rate). Conidiospores were recovered with 10 mL of sterile distilled water with 0.05% (*v*/*v*) Tween 80 using a Digralsky stick. The obtained suspension was filtered in a sterile Büchner funnel with a pore size of 45 μm (LABBOX, SL, Mataró, Barcelona, Spain).

Soy milk samples were inoculated by transferring 10 mL of the pure culture suspensions of *E. coli* and *L. monocytogenes* and 1 mL of pure spore suspensions of *B. cereus* and *A. niger* to 1 L of sterile commercial soy milk to achieve a final concentration above 10^5^ CFU/mL of vegetative cells/spores.

### 2.7. Microbiological Analysis of the Samples and Lethality Determination

Treated samples were collected aseptically in sterile containers in a biosafety cabinet (Telstar PCR Mini-V, Terrassa, Spain) and kept until analysis at 4 °C. Phosphate-buffered saline solution (PBS) (0.24 g of KH_2_PO_4_ in 1 L of distilled water, pH adjusted to 7.4; Panreac) was used to prepare tenfold dilutions from each sample. Dilutions were plated in Petri plates with trypticase soy agar enriched with 0.6% yeast extract (TSA-YE, Oxoid). Plates were incubated at 37 °C for 24 h.

The lethality caused by the UV-C treatments on the inoculated microorganism was estimated with the formula:

(6) $$\mathrm{Lethality}=-{Log}_{10}\left(\frac{N}{N_{0}}\right)$$

where *N*_0_ is the initial number of vegetative cells or spores present in the samples before the treatments and *N* is the number of remaining viable cells or spores after the treatments, both expressed as CFU/mL.

### 2.8. Determination of the Inactivation Kinetics and Effect of Treatment Variables

Survival data of vegetative cells or spores after the different UV-C treatments (T1, T2, and T3) were adjusted to different linear and nonlinear models of survival curves using the GInaFiT 1.6 software, a freeware add-in for Microsoft^®^ Excel 365 version 2309 [28]. The suitability of the adjustment was evaluated by determining the root mean square error (RMSE) value, the adjusted R squared (adjusted R^2^), and the Akaike information criterion (AIC) value. The 4D_UV-C_ value, that is, the UV-C dose necessary to reduce 4 log CFU/mL of the initial load of the microorganisms tested, was determined from data that showed the best adjustment to the models.

From the results obtained with the different matrices after the UV-C treatments (T1, T2, and T3), different statistical models were elaborated based on the lethality as a response variable and different independent variables: (1) Log C: the amount of microorganisms inoculated, expressed as log CFU/mL; (2) UV-C: dose of UV-C radiation, expressed in J/mL; (3) treatment flow rate, expressed in mL/s; and (4) NET, expressed as its absolute value. Five different models were elaborated: Model 1 presents the effect of the combination of variables of dose and flow rate; Model 2 presents the influence of the NET value with a linear adjustment; Model 3 presents the influence of NET from a quadratic approach; Model 4 included the variables of UV-C dose, flow rate, and NET with a linear adjustment; and Model 5, a quadratic adjustment using the variables dose, flow rate, and NET. The suitability of the adjustment was evaluated by determining the root mean square error (RMSE) and R squared (R^2^) values.

### 2.9. Cleaning and Disinfection of the UV Equipment

Prior to each treatment, the equipment was disinfected by recirculating a 0.1% sodium hypochlorite solution for 10 min at 20 °C. Then, 10 L of sterile softened water was circulated to remove any residual disinfectant. After the treatments, the circuit was cleaned by recirculating a nonfoaming alkaline soap solution called Capture-VC16 (Diversey España, Viladecans, Spain) at 60 °C for 15 min to eliminate any remaining debris. Subsequently, 10 L of water was circulated to rinse off the soap residue, and the circuit was then disinfected by circulating a 0.1% sodium hypochlorite solution for 10 min at 20 °C. Finally, any remaining traces of disinfectant were eliminated by pumping 10 L of sterile softened water. Before each treatment, a sample of the rinsing water was microbiologically analyzed to ensure that the equipment used during the treatments did not contribute to contamination.

### 2.10. Statistical Analysis of the Results

For each experiment, three independent trials were performed, with two different samples from each trial (*n* = 6). To compare results between treatments and/or matrices, analysis of variance (ANOVA) and Tukey test were used, considering differences significant when *p* < 0.05. This analysis was performed with the R system for statistical computation (R Foundation for Statistical Computing, Vienna, Austria 2014, http://www.R-project.org.

## 3. Results and Discussion

### 3.1. Effect of UV-C Treatments on the Inoculated Microorganism

**Figure 2:**
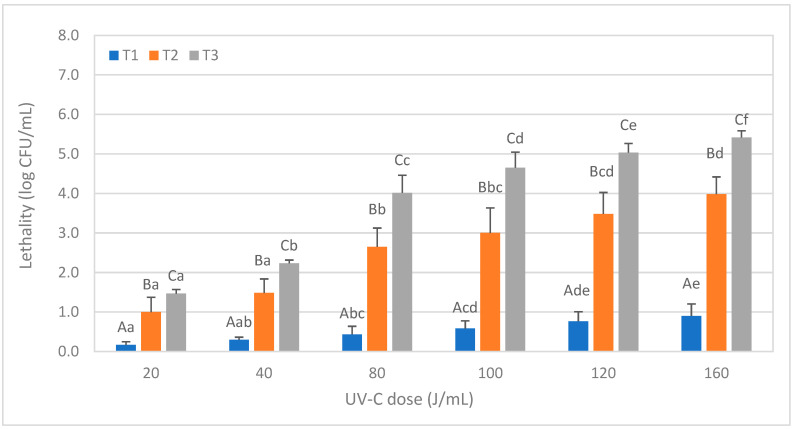
Lethality of *Bacillus subtilis* spores in soy milk caused by different ultraviolet radiation treatments consisting of different doses (J/mL) and numbers of passes (T1, T2, and T3). Results are expressed in CFU/mL ± standard deviation (SD). Different lowercase letters in the data columns indicate significant differences (*p* < 0.05) between treatments with the same number of passes (T) but different UV-C doses. Different capital letters indicate significant differences (*p* < 0.05) between treatments with a different number of passes but the same dose of UV-C.

Figure 2 shows the lethality obtained in the spores of *Bacillus subtilis,* depending on the treatment conditions tested. After T1 treatments, lethality increased significantly with the UV-C dose (*p* < 0.05), but maximum lethality was always less than 1 log. The lethal effect for the same UV-C doses increased significantly when the NET was increasing (T2 and T3 treatments), and reductions of 5 log or above were achieved only after T3 treatments at the highest UV-C doses (120 J/mL and 160 J/mL).

Bandla et al. [17] evaluated the effect of UV-C on *B. cereus* spores inoculated in soy milk and reported lethality values of 3.22 log after a UV-C dose of 11.2 mJ/cm^2^, while in this study, reductions of 1.47 log were reported for a dose of 20 J/mL, equivalent to 2082.8 mJ/cm^2^ in T3 treatment, and 4 log for a dose of 80 J/mL (equivalent to 8323 mJ/cm^2^). These differences could be attributed partly to the low AC_254_ of the soy milk reported by these authors (163 cm^−1^) in front of the 500 cm^−1^ of the soy milk of this survey. The type of reactor used by these authors (coiled tube reactor with 1.6 mm and 3.2 mm internal diameter) could be another significant factor, as it generated higher Reynolds number (Re) values than the annular thin film reactor used in this study.

**Figure 3:**
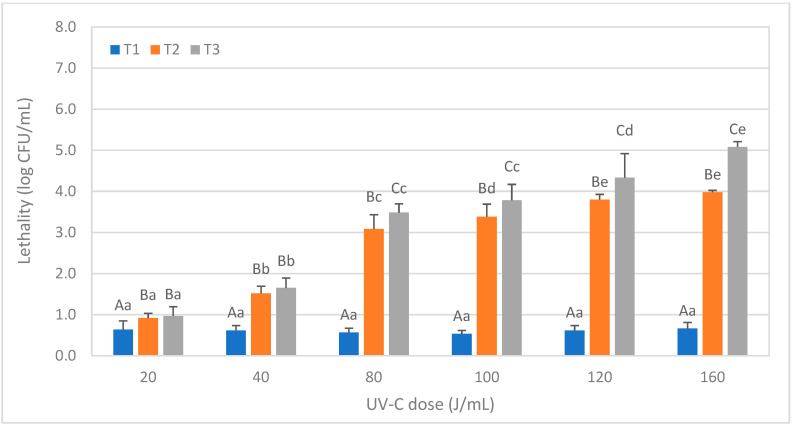
Reduction levels of conidiospore counts of *Aspergillus niger* inoculated in soy milk caused by different ultraviolet radiation treatments consisting of different doses (J/mL) and numbers of passages (T1, T2, and T3). The results are expressed as the logarithm of the initial counts minus the final counts, expressed in CFU/mL ± SD. Different lowercase letters in the data columns indicate significant differences (*p* < 0.05) between treatments with the same number of passages (T) but different UV-C doses. Different capital letters indicate significant differences (*p* < 0.05) between treatments with a different number of passes but the same dose of UV-C.

For *Aspergillus niger* conidiospores (Figure 3), no differences were observed for T1 treatments at any dose, where lethality was always below 1 log CFU/mL, but T2 and T3 significantly increased the lethality concerning T1. Nevertheless, significant differences between T2 and T3 (*p* < 0.05) were observed only after applying a dose equal to or greater than 80 J/mL. *A. niger* conidiospores showed to be more resistant than *B. cereus* spores, as a mean lethality of 5 log CFU/mL was obtained only at the highest UV-C dose tested (160 J/mL).

No previous references were found on the effect of UV-C treatments on *A. niger* in soy milk. In a study by Reverter-Carrión et al. [22], using the same reactor, a reduction of approximately 4 log CFU/mL of spores was observed in PBS with caramel dye (AC_254_ of 26 cm^−1^) after a dose of 14.3 J/mL. However, in soy milk, a dose of 160 J/mL was required for a similar reduction in T2 treatments, and 120 J/mL was needed for T3 treatments. In other kinds of matrices, Engin and Karagul Yuceer [29] reported a 3.78 log reduction in mold and yeast counts in bovine milk after applying a dose of 13.87 J/mL. Similarly, Corrales et al. [14] achieved a 3 log reduction in molds and yeasts in tiger nut milk with a dose of 4.23 J/cm^2^. In contrast, in this survey, an 80 J/mL dose was necessary with the T2 treatment to achieve a similar reduction of *A. niger* conidiospores in soy milk. These differences could be attributed to variations in the absorption coefficients of the matrices and the inherent resistance of *A. niger* conidiospores to UV-C due to their pigmentation (melanin) [30,31,32].

**Figure 4:**
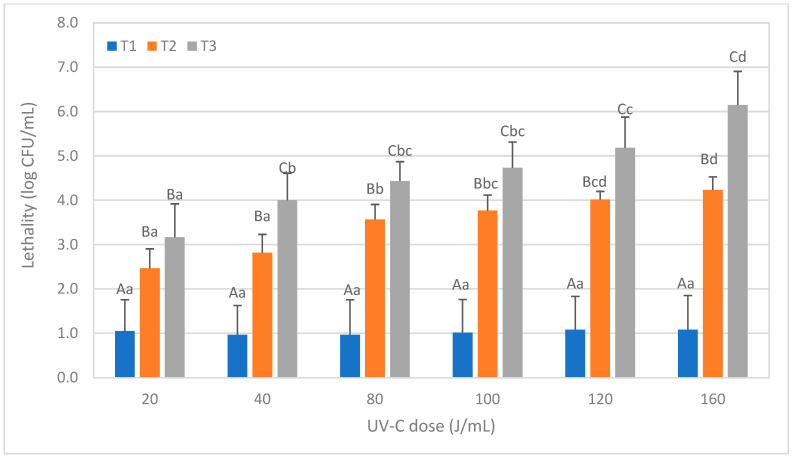
Reduction levels of inoculated *Escherichia coli* counts in soy milk caused by different ultraviolet radiation treatments consisting of different doses (J/mL) and number of passages (T1, T2, and T3). The results are expressed as the logarithm of the initial counts minus the final counts, expressed in CFU/mL ± SD. Different lowercase letters in the data columns indicate significant differences (*p* < 0.05) between treatments with the same number of passages (T) but different UV-C doses. Different capital letters indicate significant differences (*p* < 0.05) between treatments with a different number of passes but the same dose of UV-C.

A similar trend to the previous microorganisms was observed after treating the soy milk inoculated with *Escherichia coli* (Figure 4). The lethality significantly increased with T2 treatments compared to T1 (*p* < 0.05), and further increased with T3 compared to T2 at all UV-C doses except the lowest one (20 J/mL). The maximum lethality, exceeding 6 log, was obtained with the T3 treatment at a dose of 160 J/mL. However, with a dose of 120 J/mL, the lethality was already 5 log.

In a study by Bandla et al. [17] using the same strain of *E. coli*, a 5 log reduction in soy milk was achieved at a dose of 11.19 mJ/cm^2^. However, in our study, a higher dose of 120 J/mL (equivalent to 12,482 mJ/cm^2^) was required to achieve a 5 log CFU/mL reduction with the T3 treatment, although a dose of 20 J/mL (equivalent to 2083 mJ/cm^2^) was sufficient to exceed a 3 log reduction. The observed differences can be mainly attributed to variations in AC_254_ and the type of reactor used, as previously discussed for B. *subtilis.* Additionally, in the study by Orlowska et al. [33], soy milk with an AC_254_ value of 162.1 cm^−1^, inoculated with a different strain of *E. coli*, resulted in a reduction of 0.5 log with a dose of only 4.45 mJ/cm^2^ after ten passes through a UV-C reactor with laminar flow. A similar number of passes (15) was proposed for the T2 treatment, but a higher final dose of 20 J/mL (equivalent to 2083 mJ/cm^2^) was required to achieve a 2.47 log CFU/mL reduction. These differences can be attributed to both the different *E. coli* strains used and the lower AC_254_ value of the matrix.

The efficiency of UV-C to inactivate strains of *E. coli* has also been evaluated in other matrices, such as cow milk. Choudhary et al. [34] used the same *E. coli* strain as this study and compared UV-C reactors with different internal diameters (1.6 mm and 3.2 mm, with a laminar flow) and reported higher reductions in *E. coli* with the smallest internal diameter, achieving a 4.1 log reduction in whole milk and a 7.8 log reduction in skimmed milk at a dose of 11,187 mJ/cm^2^. In another study by Lu et al. [35], the impact of internal diameter (1.5 mm, 2 mm, 3 mm) on UV-C treatment effectiveness in whole milk (AC_254_ not reported) was also investigated, obtaining a 6 log reduction in *E. coli* with a dose of 20.8 mJ/cm^2^ using the smallest diameter (the sample was recirculated more than 20 times, and Re was greater than 2500). However, when the diameter was expanded to 3 mm, reductions dropped to 4 log. In this survey, to exceed a 6 log reduction, it was necessary to apply a dose of 160 J/mL, equivalent to 16,646 mJ/cm^2^, using a reactor with a smaller internal diameter (1 mm). However, according to Murakami et al. [15], the strain of *E. coli* used in our survey is more resistant to UV-C than the one used in the study by Lu et al. [35].

Some studies have evaluated UV-C treatments with strains of *E. coli* O157:H7. Crook et al. [6] inoculated whole milk with *E. coli* O157:H7 and achieved a 5 log reduction using a dose of 1.5 J/mL, but with a Re value exceeding 7500. Yin et al. [36] applied an irradiating dose of 15.9 mJ/cm^2^ through a Petri dish in inoculated pasteurized whole milk with an AC_254_ value of 476 cm^−1^, obtaining a 2.95 log reduction in *E. coli* O157:H7. According to these results, *E. coli* O157:H7 strains would be more sensitive to UV-C treatments since lower doses are required to achieve greater inactivation than *E. coli* K12 or analogous strains commonly used in UV-C radiation studies in food [17,34].

**Figure 5:**
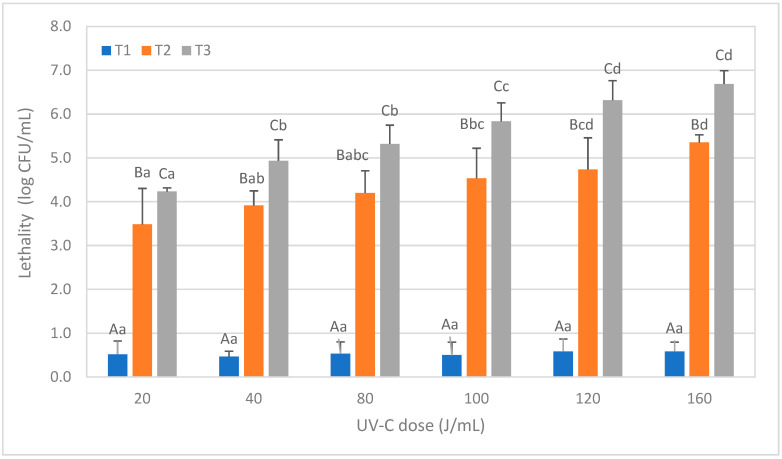
Reduction levels of inoculated *Listeria monocytogenes* counts in green tea caused by different ultraviolet radiation treatments consisting of different doses (J/mL) and number of passages (T1, T2, and T3). The results are expressed as the logarithm of the initial counts minus the final counts expressed in CFU/mL ± SD. Different lowercase letters in the data columns indicate significant differences (*p* < 0.05) between treatments with the same number of passages (T) but different UV-C doses. Different capital letters indicate significant differences (*p* < 0.05) between treatments with a different number of passes but the same dose of UV-C.

The effect of the UV-C radiation treatments on the lethality of *Listeria monocytogenes* is summarized in Figure 5. As observed with *E. coli,* T1 treatments did not reach a 1 log reduction at any tested dose. However, with T2 treatments, lethality gradually increased, and a reduction higher than 5 log was achieved at a dose of 80 J/mL. The maximum lethality of 6.68 log was reached with the T3 treatment at a dose of 160 J/mL.

To date, no published results are available regarding the impact of UV-C treatment on soy milk inoculated with *L. monocytogenes*. However, studies have been conducted and reported on other matrices with a high absorption coefficient. Matak and Churey [37] achieved an effective reduction of 5.62 log in fresh whole goat milk by applying a cumulative dose of 15.8 mJ/cm^2^ after 12 passages through the reactor with a Re value of 5187. Similarly, Crook et al. [6] obtained the same lethality in whole bovine milk with a dose of 2.0 J/mL, but with a Re value exceeding 7500. On the contrary, Lu et al. [35] and Pereira et al. [38] reported lower lethality values, below a 4 log reduction, in whole cow’s milk, even with total UV-C radiation doses of 21.7 mJ/cm^2^ after 20 passages through the reactor with a Re value exceeding 2500 in the first case, and a dose of 1350 mJ/cm^2^ after 120 passages in the second. In this study, a dose of 40 J/mL, equivalent to 4165.5 mJ/cm^2^, was required to achieve a 5 log reduction in *L. monocytogenes*. In another investigation by Elbrhami [39], UV-C treatments on *Listeria innocua* inoculated in tiger nut milk, with a lower AC_254_ value of 193 cm^−1^, resulted in 5 log reductions after applying a dose of only 17.9 J/mL through five cycles or passages. Similar results were obtained in this study with the T3 treatment at 20 J/mL for *L. monocytogenes*, which reduced the initial load by 4.25 log. Once again, various factors, including the strain used, reactor designs, and AC_254_ of the matrices (information not always provided), are possible explanations for the significant differences in reported effectiveness.

### 3.2. Characterization and Evaluation of the UV-C Reactor

The results were significantly influenced by the functional characteristics of the UV-C reactor. One of the main factors to consider was the distance the UV-C light had to cross through the liquid food matrix, which was 1 mm in this case. Although this short distance should increase the UV-C radiation exposure of all liquid food matrices, generating a turbulent flow is impeded, even when high flow rates are applied. As shown in Table 1, even in treatments with the highest flow rate (T3), the maximum Reynolds number (Re) reached was only 1015, indicating laminar flow (<2400). Turbulent flow is of particular relevance in the effectiveness of UV-C in matrices with a high AC_254_ since it increases the probability of all the particles of the matrix reaching the area where the lethal effect of UV-C is maximum [17,19,34]. Considering the limitation of creating turbulent flow in this reactor, one approach to increasing the probability of particles reaching the lethal zone is by recirculating the matrix through the reactor. The UV-C dose received then depends on the flow rate and the number of passes through the reactor. The number of entries and exits of any particle (or microorganism) in the liquid food matrix is defined as the NET value. For example, in T2 treatments, the product received a dose of 160 J/mL when recirculated for 48 min at a flow rate of 41.87 mL/s supposed 241 inputs and outputs, while in T3 treatments, where the flow rate was 64.6 mL/s, the number of inputs and outputs (NET) necessary to achieve the same UV-C dose was 372. As the number of passes increases, the probability that the microorganisms are in the UV-C most effective area (lethal zone) also increases. The calculated depth of the lethal zone for soy milk was 0.032 cm, corresponding to a 3.2% probability of a particle being in the lethal zone. With these data, the NEL value, defined as the number of times any particle passes through the lethal zone, can be estimated. For example, for T1, T2, and T3 treatments at a UV-C dose of 160 J/mL, where the NET values were 2, 241, and 372, respectively, the NEL values obtained were 0.064, 7.72, and 11.9. Based on these results, the NET value would be critical to evaluate lethality achieved on high AC_254_ matrices. The higher the NET value, the higher the number of times each particle or volume portion gets into the zone of maximal germicidal efficacy (NEL).

### 3.3. Mathematical Modelling of UV-C Treatments

Five linear or quadratic regression models were developed, based on the variables or factors that can be determined to explain microbial lethality, such as the UV-C dose, the flow rate, and the NET value. The initial concentration of the microorganism (Log_c_) was also included as a covariate. The five models evaluated were Model 1: linear dose–flow rate; Model 2: linear NET; Model 3: quadratic NET; Model 4: linear dose–flow–NET; and model 5: quadratic dose–flow–square NET. Different statistical parameters were considered to choose the best model where the predicted values were as close as possible to the observed data, prioritized in the following order: (1) the R^2^ as close as possible to 1.0; (2) the root mean square error (RMSE)^2^ as close as possible to 0, indicating a good fit of the data to the model; and (3) the simplicity of the model (model with the smaller number of variables). Table 2 shows the statistical parameters obtained for the five models evaluated, and Table 3 shows the adjustment of the selected models for each microorganism according to the abovementioned criteria.

In the case of *A. niger* conidiospores, model 3 was chosen over model 5, although R^2^ and RMSE were slightly better for model 5. Model 3 had just three variables (LogC, *NET*, and *NET*^2^), and adding the variables of dose and flow rate (model 5), the adjusted R^2^ only improved by 0.01 and the RMSE by 0.05. In general, it can be concluded that the number of passes (*NET* value) is the most relevant factor in the selected models showing statistically significant differences (*p* < 0.05) regarding the applied dose. A previous survey by Martínez-García et al. [13] obtained similar results in whole and skimmed cow’s milk for *B. subtilis.*

### 3.4. Inactivation Kinetics and Calculation of the 4D_uv-c_ Value for UV-C Treatments

In addition to the determination of the previously evaluated mathematical models of inactivation, survival data of the tested microorganisms after the different UV-C treatments (T2 and T3) were adjusted to different nonlinear survival curves using GInaFiT software [28]. No models were evaluated in the T1 treatments because no differences were observed at different doses. Models were selected using the best fit precision parameters as the lowest Akaike information criterion (AIC), RMSE close to 0, and the adjusted R^2^ close to 1 [40] (Table 4).

**Figure 6:**
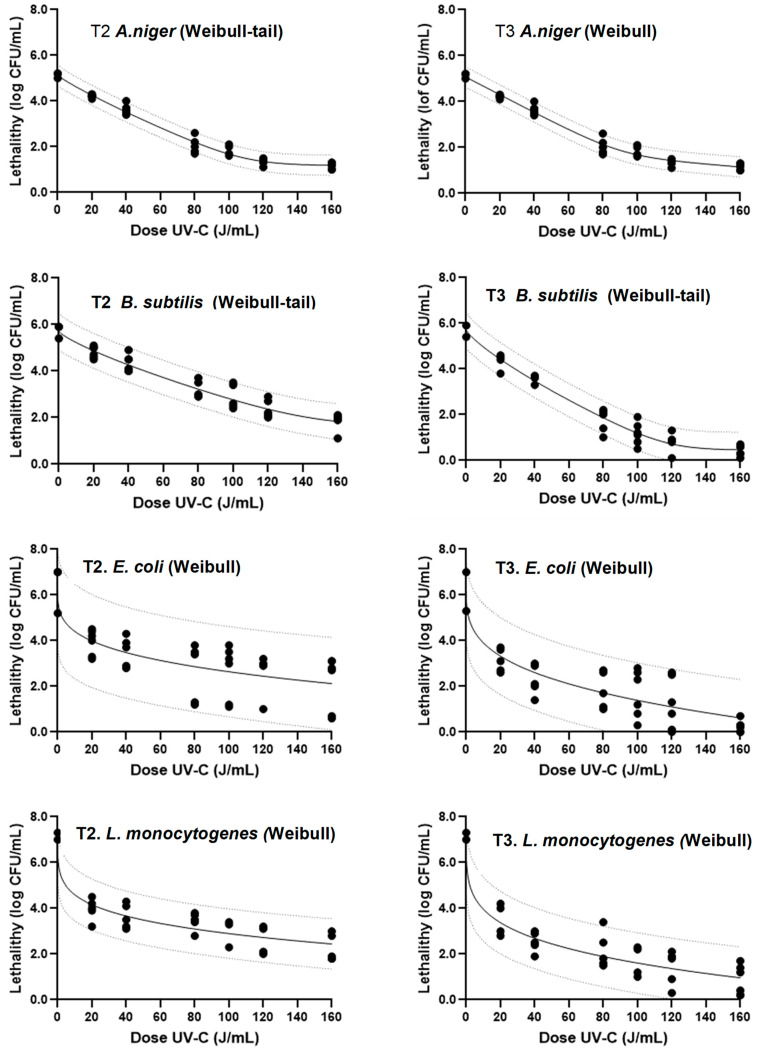
Adjustment of the data of surviving spores of *Aspergillus niger* and *Bacillus subtilis*, and vegetative cells of *Escherichia coli* and *Listeria monocytogenes* to the Weibull inactivation model (with and without tail) using the tool GInaFIT after T2 (left column) and T3 (right column) treatments at different UV-C doses. Black circles correspond to the data points, the solid line shows the estimated curve according to the model and the dotted lines the prediction bands.

Inactivation models with better fit were nonlinear and adjusted to a Weibull and Weibull tail model proposed by Marfart et al. [41] and by Albert and Mafart [42], respectively (Table 4 and Figure 6). The presence of tails in the data indicates that a bacterial population showed greater resistance regardless of the increase in dose. The tailing effect is caused by inadequate mixing of the food liquid matrix, the formation of clumps of organisms, or their attachment to the (comparatively dark) surface of the product [43]. This phenomenon was observed in *B. subtilis* in the T2 and T3 treatments and *A. niger* in the T2 treatment (Figure 6). The δ value represents the time required for the first logarithmic reduction due to UV-C action, while the *p* value represents the shape factor of the Weibull distribution. A value of *p* < 1 indicates a concave shape, suggesting that accumulated damage sensitizes the survivors, while a value of *p* > 1 indicates a convex shape, indicating the rapid elimination of weaker members and the progressive survival of more UV-C-resistant individuals [44]. The 4D_UV-C_ value, estimated using the GinaFiT tool, represents the UV-C dose required to achieve a 4 log reduction. This value is used for comparing nonlinear survival curves. Different resistance behaviors to UV-C treatments were observed among microorganisms. *A. niger* and *B. subtilis* strains showed higher δ, *p*, and 4D_UV-C_ values after applying T2 and T3 treatments, indicating their higher resistance than vegetative bacteria. A decrease in these values was observed for all microorganisms when T3 treatments were applied (Table 4). The resistance sequence from least to most resistant was *L. monocytogenes* < *E. coli* < *B. subtilis* < *A. niger*.

Some microbial models have been used to model the survival curves of microorganisms in milk, tiger nut, and soy milk products with UV-C light. Martínez-Garcóa et al. [13] reported similar results in whole cow’s milk for *B. subtilis*, in which the inactivation model was the Weibull tail with values at T2 and T3 of 94 and 91 J/mL. Croock et al. [6] also found a Weibull-type fitting model for *E. coli* and *L. monocytogenes* in milk, with *p* values greater than 1.6 and 4D values of 1.7 and 1.3 J/mL, respectively, which are lower than the results obtained in this study. In the case of tiger nut, the best fit for *E. coli* was also Weibull, with *p* values below 1 and a 4D value of approximately 20 mJ/cm^2^ [45]. However, in our study, the T2 and T3 values were significantly higher, corresponding to 13,121.64 and 5831.84 mJ/cm^2^. However, Orlowska et al. [33] found that the inactivation kinetics for this microorganism in soymilk were linear with a D-value of 6.71 mJ/cm2 (4D_UV-C_ of 26.72 mJ/cm^2^).

The intrinsic factors of the liquid foods, the type of equipment, and its flow rate and mixing characteristics, as well as the applied dose, are very important factors to obtain a well-processed product. Although the first-order approach is the most commonly used approach to understand the effect of UV-C light on microorganisms, it seems that this approach is only suitable for water and clarified products. Therefore, it is necessary to use nonlinear models to fully describe the effect of UV-C light on liquid food products [43].

## 4. Conclusions

Recirculation appears to be a good strategy for applying UV-C treatments using an annular radial UV-C reactor in matrices with high interference, such as soy milk. The mixing effect would ensure that microorganisms will be in the maximum efficacy area (where the action of UV-C radiation is the highest) for enough time to be inactivated. This hypothesis seems to be demonstrated by NET being the most determining variable for the lethality prediction of the tested microorganisms. Although pasteurization of soy milk using UV-C seems an achievable goal, commercial sterilization is complicated since the estimated 4D_UV-C_ values needed to inactivate microbial spores are too high. However, implementing correct hygiene practices in the preparation of raw materials and during treatments, or the combined application of other technologies, seeking a hurdle effect, could allow for a lethality of less than 4 Log to be sufficient to guarantee the quality of the product [13,22].

## Figures and Tables

**Table 1:** Characteristics of the treatments along with their variables.

Treatments	Flow Rate (mL/s)	*NET* ^1^	RPM ^2^	Retention Time (s)	Dose (J/mL)	Reynolds
T1	<2.9	2	43	12	10	105
			21	24	20	52
			12	48	40	26
			6	96	80	13
			5	120	100	10
			3	144	120	9
			1.5	192	160	7
T2	42	15	300	180	10	658
		30		360	20	
		60		720	40	
		121		1440	80	
		151		1800	100	
		181		2160	120	
		241		2880	160	
T3	62	23	500	180	10	1015
		46		360	20	
		93		720	40	
		186		1440	80	
		233		1800	100	
		279		2160	120	
		372		2880	160	

^1^ *NET*: number of entries in the reactor tube. ^2^ RPM: revolutions per minute.

**Table 2:** Statistical parameters of the mathematical models based on the lethality obtained in soy milk after the treatments with UV-C radiation.

Type	Model 1		Model 2		Model 3		Model 4		Model 5	
	Linear		Linear		Quadratic		Linear		Quadratic	
	*L* = *C* + *a*·Log_c_ + *b·D* + *c*·FR		*L* = *C* + *a*·Log_c_ + *b*·*NET*		*L* = *C* + *a*·Log_c_ + *b*·*NET* + *c*·*NET^2^*		*L* = *C* + *a*·Log_c_ + *b*·D + *c*·FR + *d*·*NET*		*L* = *C* + *a*·Log_c_ + *b*·D + *c*·FR + *d*·*NET* + *e*·*NET^2^*	
**Microorganism**	**R^2^**	**RMSE**	**R^2^**	**RMSE**	**R^2^**	**RMSE**	**R^2^**	**RMSE**	**R^2^**	**RMSE**
*B. subtilis*	0.88	0.62	0.93	0.46	0.95	0.39	0.95	0.40	0.96	0.37
*A. niger*	0.80	0.72	0.94	0.40	0.96	0.31	0.96	0.41	0.97	0.26
*E. coli*	0.87	0.62	0.82	0.74	0.85	0.67	0.92	0.51	0.92	0.51
*L. monocytogenes*	0.94	0.58	0.75	1.13	0.85	0.90	0.95	0.50	0.96	0.46

*L*: lethality (log CFU/mL); Log_c_: initial concentration of microorganisms (log CFU/mL); *C*: constant; *D*: dose (J/mL); FR: flow rate (mL/s); *NET*: number of entries to the tunnel.

**Table 3:** Selected mathematical models with their respective equations for soy milk and inoculated microorganisms.

Microorganism	Model	Equation Model
*B. subtilis*	3	*L* = −0.41 + 0.15·Log_c_ + 0.02·*NET* − 0.00002·*NET*^2^
*A. niger*	3	*L* = −3.03 + 0.69·Log_c_ + 0.02·*NET* − 0.00002·*NET*^2^
*E. coli*	4	*L* = −1.086 + 0.31·Log_c_ + 0.0011·D + 0.032·FR + 0.0079·*NET*
*L. monocytogenes*	4	*L* = −1.027 + 0.19·Log_c_ + 0.0018·D + 0.06·FR + 0.0068·*NET*

*L*: lethality (log CFU/mL); Log_c_: initial concentration of microorganisms (log CFU/mL); *D*: dose (J/mL); FR: flow rate (mL/s); *NET*: number of entries to the tunnel.

**Table 4:** Model adjustment parameters and 4D_UV-C_ values according to the type of treatment, microorganism, and applied model.

	*A. niger*		*B. subtilis*		*E. coli*		*L. monocytogenes*	
Treatments	T2	T3	T2	T3	T2	T3	T2	T3
Model	Weibull Tail	Weibull	Weibull Tail	Weibull Tail	Weibull	Weibull	Weibull	Weibull
δ (J/mL)	23.16	16.03	24.86	14.36	0.82	0.49	2.33	2.07
*p*	0.88	0.72	0.79	0.79	0.28	0.30	0.35	0.38
4D_UV-C_ (J/mL)	±134.4	±108.8	±156.8	±84.8	±126.4	±56	±124.8	±19.2
Adjusted R^2^	0.977	0.971	0.923	0.961	0.5682	0.784	0.860	0.8731
RMSE	0.21	0.278	0.355	0.349	0.959	0.798	0.524	0.644
AIC	−115.7	−92.2	−73.23	−74.54	2.80	−11.6	−44.29	−28.32

δ: time to achieve the first log reduction; *p*: the shape of the inactivation curve; 4D_UV-C_: UV-C dose to achieve a 4 log reduction; RMSE: root mean squared error; AIC: Akaike information criterion.

## Data Availability

Data is contained within the article.
